# Cancer Stem Cells: Repair Gone Awry?

**DOI:** 10.1155/2011/465343

**Published:** 2010-12-05

**Authors:** Fatima Rangwala, Alessia Omenetti, Anna Mae Diehl

**Affiliations:** ^1^Division of Cellular Therapy, Hematology and Oncology, Department of Medicine, Duke University Medical Center, Durham, NC 27710, USA; ^2^Division of Gastroenterology, Department of Medicine, Duke University Medical Center, Snyderman Building (GSRB-1), 595 LaSalle Street, Suite 1073, Durham, NC 27710, USA

## Abstract

Because cell turnover occurs in all adult organs, stem/progenitor cells within the stem-cell niche of each tissue must be appropriately mobilized and differentiated to maintain normal organ structure and function. Tissue injury increases the demands on this process, and thus may unmask defective regulation of pathways, such as Hedgehog (Hh), that modulate progenitor cell fate. Hh pathway dysregulation has been demonstrated in many types of cancer, including pancreatic and liver cancers, in which defective Hh signaling has been linked to outgrowth of Hh-responsive cancer stem-initiating cells and stromal elements. Hence, the Hh pathway might be a therapeutic target in such tumors.

## 1. Introduction

The cancer stem cell hypothesis assumes that cancers originate from mutated tissue stem cells (SCs) that have dysregulated self-renewal properties, and thus selectively drive tumor growth. Current chemotherapeutic agents and radiation therapy target the rapidly dividing cells that form the bulk of the tumor but do not target the relatively quiescent cancer stem cell (CSC), thus possibly accounting for treatment failures. Furthermore, both SCs and CSCs appear to have multidrug resistance, antiapoptotic machinery, and enhanced DNA repair allowing them to evade conventional treatments [1–4]. Thus, to target tumors effectively and with minimal toxicity, drugs need to be identified that specifically target the relatively rare CSC population while sparing normal tissue stem cells.

## 2. Biologic Properties of Cancer Stem Cells

The properties that CSCs are postulated to exhibit are: (1)tumorigenic capacity or self-renewal, (2)the potential for multilineage differentiation (such that they can recapitulate the multiple tumor cell types found in the parent tumor), (3)serial passage, and (4)expression of a unique repertoire of surface markers that allow for their reliable identification and purification [5]. Specifically, the CSC hypothesis posits that within a given tumor, only a distinct phenotypic subset of cells has tumorigenic capacity, that is, injection of this cell fraction into nude mice is able to fully recapitulate the parent tumor. Currently, serial passage in xenotransplantation models is the gold standard assay for defining the CSC fraction [6].

Normal tissue SCs tightly regulate the balance between self-renewal and differentiation, and quiescence and proliferation (reviewed in [7, 8]). SC number, in the context of the stem cell niche, is precisely maintained via the ratio of symmetric and asymmetric cell divisions. Dysregulation of self-renewal pathways appears to result in CSC overpopulation [9, 10]. This may be due to an increase in symmetric divisions of the CSC and may represent a potential drug target [11, 12]. 

As has been well characterized in the hematopoietic system, the cells within solid organs appear to demonstrate a hierarchy in which stem cells give rise to committed progenitor cells which give rise to rapidly proliferating cells which give rise to terminally differentiated cells. As SCs mature from a self-renewable stem cell to a terminally differentiated cell, they progressively lose their ability for self-renewal and pluripotency but gain mitotic activity. By analogy to normal tissue SCs, CSCs must able to give rise to all of the different cell types that comprise a given tumor, lending credence to the idea that tumors can be viewed as aberrantly functioning complex organs. Tumor heterogeneity is likely a consequence of ongoing accumulation of mutations over time that result in variable degrees of cellular differentiation.

## 3. Dysregulation of Self-Renewal Pathways in CSCs: Hedgehog Pathway Involvement

Mutations in fundamental self-renewal pathways, such as the Hh pathway, likely cause normal stem cells to become independent of regulatory signals that are generated by their microenvironment. These pathways play a critical role in development and are usurped by transformed cells for tumor initiation, progression, and metastasis. The hedgehog proteins are secreted signaling proteins that were initially identified in *Drosophila* as segment polarity genes. In multiple tissue types, the Hh pathway plays a key role in organogenesis, patterning, and stem cell maintenance. Depending upon context, Hh can function both as a morphogen and a mitogen. As a morphogen, Hh induces cell differentiation in a concentration-dependent manner. As a mitogen, it drives the proliferation of precursor cells and mediates the interaction between the epithelial and mesenchymal compartments [13]. In multiple tissue types, it has been demonstrated that mesenchymal cells and progenitor cells are Hh responsive. In contrast, mature epithelial cells are typically nonresponsive to Hh. 

The components of the Hh signal transduction machinery have been elucidated, and while there are some important differences across species, the pathway remains relatively well conserved between *Drosophila *and higher organisms. Three secreted Hh ligands have been identified in humans, Sonic hedgehog (Shh), Indian hedgehog (Ihh), and Desert hedgehog (Dhh) and are known to signal in an autocrine and paracrine manner. In the absence of Hh ligand, Patch (PTCH), the hedgehog receptor binds and inhibits Smoothened (SMO) and thus prevents target gene transcription. Binding of the Hh ligand to PTCH releases SMO and allows for further propagation of the signal, leading to target gene transcription through three Gli transcription factors, Gli1, Gli2, and Gli3. Gli proteins regulate target gene expression by direct association with a consensus sequence located within the promoter of the target genes. Thus, PTCH is a negative regulator and SMO a positive regulator of the pathway [14]. Intriguingly, in mammals, it appears that protein components of the primary cilium are required for the coordination of hedgehog signaling [15, 16]. 

Loss of function mutations in the Hh receptor, PTCH, were initially noted in patients with Gorlin syndrome or basal cell nevus syndrome [17, 18]. Gorlin patients have a variety of developmental defects and have an increased incidence of benign and malignant tumors including basal cell carcinomas (BCC), medulloblastomas, ovarian fibromas, rhabdomyosarcomas, and meningiomas. A large majority of sporadic medulloblastomas and basal cell carcinomas also exhibit hyperactive Hh signaling (in a ligand-independent fashion) due to mutations in Hh pathway components including, PTCH, SMO, and occasionally Suppressor of Fused (SUFU), a negative regulator of the Hh pathway [19]. In clinical trials, SMO inhibitors have now been demonstrated to have potent antitumor activity in patients with BCC and one patient with medulloblastoma, thus confirming an “oncogene addiction” model in which deregulated Hh signaling results in increased cell proliferation and tumor formation [20, 21].

There is now a growing body of evidence that the Hh pathway is activated in a variety of other solid tumors including colorectal, pancreatic, breast, and small cell lung cancer and hepatocellular carcinoma [22–28]. Unlike BCC and medulloblastoma, Hh activation in these tumor types is not mutation driven. Both in vitro and in vivo, Hh pathway activation appears to impact tumor progression via effects on cell proliferation, cell survival, angiogenesis, invasion, and genetic instability. Given that this data has been reviewed recently and comprehensively [14, 19, 29], we will focus on the role of Hh signaling in cancer stem cell expansion and maintenance with a focus on pancreatic cancer and hepatocellular carcinoma (HCC).

Increased Hh pathway activity was first documented in early pancreatic cancer by Thayer et al. where the degree of Shh expression in the ductal epithelium was noted to positively correlate with degree of atypia [23]. While Shh expression appeared to be confined to the epithelium, increased PTCH expression was seen in the abnormal epithelium and the surrounding mesenchymal cells suggesting both an autocrine and paracrine mechanism of signaling. Furthermore, treatment of pancreatic cancer cells lines with the SMO inhibitor, cyclopamine, resulted in decreased cell proliferation and increased apoptosis further supporting autocrine signaling. In vivo, cyclopamine treatment of pancreatic tumor xenografts resulted in decreased tumor mass suggesting an important role for Hh signaling in cell survival and proliferation. 

A series of experiments that have been performed to further define an epithelial versus stromal role for Hh signaling have led to contradictory results. Yauch et al. demonstrated that in both colon and pancreatic adenocarcinomas, epithelial tumor cells secrete high levels of Hh ligand in comparison to normal tissue controls. Hh pathway activation is noted in the neighboring stromal cells but not in the epithelial cells consistent with a paracrine signaling mechanism [30]. In addition, expression of an oncogenic allele of SMO in the pancreatic epithelium did not result in Hh pathway activation and had no impact on the development of pancreatic neoplasia, while SMO activation in the mesenchyme led to Hh pathway activation. Taken together, these data suggest that only the stromal compartment is competent to activate Hh signaling [31]. While this data is consistent with Hh signaling in CSCs, which do not express markers of epithelial differentiation, these results conflict with considerable evidence for ligand-dependent Hh-signaling in epithelial cells of multiple other tumor types [32]. These discrepancies may be explained in part by differing mouse models, human tumor xenografts versus transgenic models of spontaneous carcinomas. Clearly, further study is required to define the precise mode of Hh signaling, as it will have important ramifications for drug development.

In order to determine whether increased Hh activity is a property of all pancreatic tumor cells or is selective for the CSC, Li et al. demonstrated, using quantitative real time-pcr, that Shh transcripts were 4-fold upregulated in the bulk pancreatic xenograft cells and 43-fold upregulated in the CD44+CD24+ESA+ pancreatic CSC as compared with normal pancreatic epithelial cells [33]. Active Hh signaling has now also been identified in the CSCs from glioblastoma and breast tumors and modulation of Hh pathway activity in these cell types results in decreased tumorigenic potential and depletion of the CSC compartment [34–36].

## 4. Tissue Injury and Repair: A Potential Source of CSCs

In development, both the pancreas and the liver are specified by domains of the gut endoderm. Embryonic outgrowths of the dorsal and ventral regions of the foregut endoderm give rise to the endocrine and exocrine pancreas. The liver, however, derives solely from the ventral foregut endoderm [37, 38]. Using embryo tissue explant assays, it has been shown that the default fate of the ventral endoderm is to activate the pancreatic organogenesis program. In the developing embryo, the ventral foregut becomes closely apposed to the cardiac mesoderm, and this proximity is required for liver specification [38]. FGF signaling from the cardiac mesoderm induces the local production of sonic hedgehog, and BMP signaling from the underlying septum transversum mesenchyme cells coordinately directs the development of the liver bud and inhibits pancreas development. Conversely, treatment with the BMP inhibitor, noggin, enhances pancreatic gene expression and suppresses hepatic gene expression in ventral foregut explants [39, 40]. These multiple signaling pathways direct endoderm patterning via induction of specific transcription factors. The Forkhead Box A proteins (Foxa) are transcription factors expressed in endoderm derived tissues and are required for hepatic, pancreatic, and pulmonary specification [41]. Transcriptional activation of foxa2 is directly regulated by the hedgehog target genes, gli proteins [42], providing support for its central role in hepatic organogenesis. The liver bud then gives rise to cells destined to become bipotential liver progenitor cells, or hepatoblasts, which give rise to two distinct lineages, hepatocytes and cholangiocytes [41]. 

A comparison of pluripotent, murine embryonic stem (ES) cells, endodermally lineage-restricted ES cells, and mature hepatocytes suggests that hedgehog activity is progressively silenced as progenitors differentiate into mature liver parenchymal cells [43]. Treatment of hepatic progenitors isolated from human livers (EpCAM+ cell fraction) with cyclopamine results in increased cellular necrosis and apoptosis, demonstrating that Hh activity is required for optimal viability of the progenitor cells. While minimal Hh ligand production is seen in healthy, human hepatocytes, the small resident progenitor population within the normal adult liver, residing along the canals of Hering, continues to demonstrate expression of Hh ligands, and Hh-responsive target genes [44, 45].

**Figure 1:**
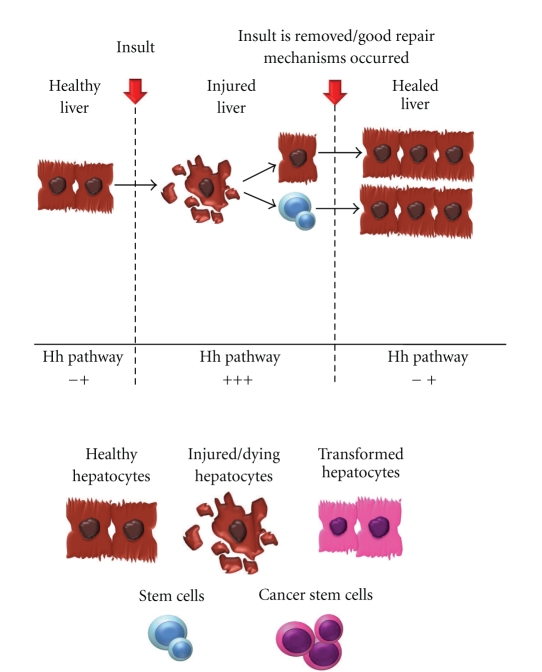
Healthy adult livers exhibit little hedgehog activity. In the presence of liver injury, hepatocytes are subject to cellular stress and in some cases undergo apoptosis. In this setting of injury, hedgehog ligand production increases resulting in the appropriate expansion and differentiation of progenitor cells allowing for liver reconstruction. As the insult is removed, Hh pathway activity slowly declines, and the progenitor population gradually dwindles away with recovery of liver health.

Hh signaling is reactivated in the liver after acute or chronic liver injury when major reconstruction of the adult liver is required. Moreover, the degree of hepatic production of Hh ligands and accumulation of Hh responsive cells is typically proportionate with the severity of liver damage and fibrosis. Immunohistochemical analysis of diseased human livers, such as those from patient with chronic viral hepatitis [46], alcoholic liver disease [47], and nonalcoholic fatty liver disease [44, 48], confirms that Hh signaling and the number of Hh responsive cells is increased in the presence of injury. The Hh-responsive cells are noted in the expanded progenitor and stromal cell compartments, suggesting that these cells actively contribute to regenerative processes in adult livers. This was confirmed by treating healthy, adult mice with cyclopamine after 70% partial hepatectomy, which provides a tremendous regenerative pressure. In comparison to vehicle control, inhibition of Hh signaling after partial hepatectomy prevented normal liver regeneration by blocking progenitor expansion and fibrogenic liver repair. Notably, cyclopamine-treated animals were unable to reconstitute their liver mass, and there was significant mortality in the group 48-hours postpartial hepatectomy in comparison to vehicle treated controls [49]. Furthermore, in rodent models of liver injury, once the offending agent is removed, Hh ligand levels decline and Hh-responsive cells gradually regress as normal liver architecture is restored [50, 51]. Taken together, these data suggest that in the setting of acute injury, transient activation of the Hh pathway is necessary for effective regeneration of the adult liver (Figure 1). However, chronic or persistent injury results in prolonged increases in Hh signaling, thus perpetuating the expansion of myofibroblasts and liver progenitor cells which contribute to fibrotic repair or cirrhosis. 

This data has significant implications for the development of HCC as cirrhosis is the most important determinant of risk for the development of HCC. Hh signaling activates prosurvival pathways in several types of liver cells including malignant hepatocytes. Expression of Shh is noted in approximately 60% of HCCs and expression of the Hh target genes, Gli1 and PTCH, was noted in 50% of tumors suggesting that the Hh pathway is activated in HCC. In three HCC cell lines, Hep3B, Huh7, and PLC/PRF/5, with detectable expression of Hh target genes, treatment with Hh neutralizing antibodies or cyclopamine resulted in cell growth inhibition and apoptosis. No effects on cell viability were noted in the two HCC cell lines that lacked Hh signaling, demonstrating that the results were specific to the Hh pathway [27, 28]. 

In multiple tissue types, Wnt pathway activity is also crucial to the maintenance of the stem cell compartment and a complex interaction of Wnt, Notch, Hedgehog, and Bmp signaling regulates the balance between stem cell renewal and cellular differentiation [52, 53]. While the role of Wnt signaling in liver SCs remains poorly understood, prior work in a mouse model of chronic liver injury has demonstrated that Wnt ligand induction is localized to postnatal hepatic progenitors both in vitro and in vivo [54]. Abnormal regulation of the Wnt signaling pathway has been extensively described as a key early carcinogenic event in HCC development. Multiple groups have demonstrated that HCCs harbor activating mutations of *β*-catenin, a transcription factor in Wnt signaling, or inactivating mutations of AXIN1 and AXIN2, negative regulators of this pathway [55–60]. Furthermore, treatment with anti-Wnt-1 antibodies dose dependently inhibits the viability and proliferation of Wnt-1 overexpressing HCC cell lines, Huh7 and Hep40, but not normal hepatocytes. Intratumoral injection of anti-Wnt-1 antibody also suppresses tumor growth in a Huh7 xenograft model via induction of tumor cell apoptosis [61]. Further studies will be required to clarify the role of Wnt signaling in liver CSCs. 

**Figure 2:**
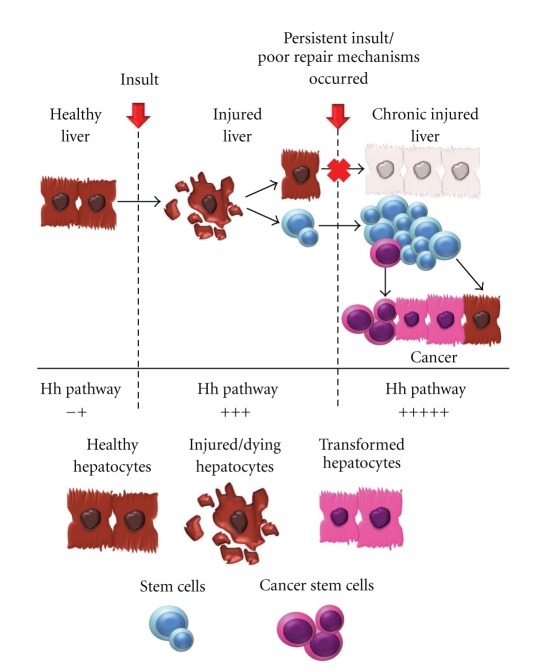
In the presence of continued liver injury, there is ongoing upregulation of the Hh pathway and persistent expansion of the progenitor pool. Thus, there is an increased probability for the accumulation of genetic or epigenetic events in the tissue stem cells resulting in oncogenic transformation to a cancer stem cell, and ultimately leading to tumor initiation.

Taken together, these data suggest that stem cell renewal pathways such as Hh and Wnt are upregulated in the setting of chronic liver injury and result in the expansion of liver progenitor populations. Ongoing production of ligand confers a survival advantage to these immature cells, some of which may eventually become tumor-initiating cells for primary liver cancers [62, 63]. This model of cancer in which neoplasia is the result of overexuberant tissue repair is supported by the strong association between chronic tissue injury and cancer incidence [64]. This association is particularly robust in hepatobiliary cancers, where exposure to a variety of toxins [65, 66], chronic infections [46, 67, 68], and inflammatory conditions [69, 70] significantly increases cancer risk. In the setting of ongoing injury, expansion of the progenitor pool persists allowing for the accumulation of genetic or epigenetic events that constitutively activate the stem/progenitor cell compartment, thus resulting in tumor initiation (Figure 2).

Investigators have now identified multiple markers that have been used to successfully enrich the CSC fraction from HCCs [52, 71, 72]. Genetic alterations in self renewal and pluripotency pathways, including Wnt/*β*-catenin [73], Notch [74], Hh [74], myc [75], and TGF-*β* [52], have been documented in these cells. Furthermore, epigenetic changes including alterations in DNA methylation patterns [76] and the aberrant expression of both oncogenic [77, 78] and tumor suppressive [79] microRNAs have been shown to contribute to hepatocarcinogenesis. These results beg the question: would targeting the tissue SC be an effective chemoprevention strategy?

## 5. Multiple Roles of Cancer Stem Cells: Pancreatic Cancer as a Model

Ontologically, a stem cell-like cell is an ideal target for transformation due to their capacity for self-renewal and long life. Pathways such as Hedgehog, Notch, and WNT have well established roles in organogenesis and normal stem cell renewal (reviewed in [13, 80, 81]). Growing evidence suggests that these pathways are dysregulated in transformation and thus contribute to tumor initiation, tumor maintenance, and metastasis of multiple tumor types [22, 23, 82–86]. Furthermore, as a consequence of self-renewal, stem cells persist for long periods of time, likely allowing them to accumulate the numerous mutations necessary for transformation [87]. But the ultimate origins of CSCs remain controversial at this time. Whether CSCs arise from existing tissue stem cells, restricted progenitor cells, or from mutations in terminally differentiated cells, resulting in dedifferentiation likely depends on context (reviewed in [8, 88]). Nonetheless, each of these scenarios is consistent with the idea that distinct population of cells with stem cell properties is essential for the development and perpetuation of various human cancers.

Utilizing a xenograft model of primary pancreatic adenocarcinoma, Li et al. purified a subpopulation of CD44+CD24+ESA+ pancreatic cancer cells (approximately 0.2%–0.8% of the total cell number) that demonstrated the requisite CSC properties of self-renewal, tumorigenic capacity, and production of phenotypically diverse progeny [33]. Combinatorial approaches demonstrated that the cells expressing all three markers had the highest tumorigenic potential, and the percentage of cells expressing all three markers remained constant with serial passage. These three markers have been previously used to mark CSCs in other tumor types and are known to act as cell adhesion molecules with multiple signaling functions (reviewed in [6]). 

However, the role of CSCs may not be confined to tumor initiation and growth, but may also play a role in metastases. Previous studies had demonstrated that in colon cancer and brain tumors, CD133+ marks the CSC population [9, 10, 89]. Hermann et al. demonstrated that the tumorigenic potential of pancreatic cancer is restricted to CD133+ cells, as they recapitulate the original tumor when injected into athymic mice [90]. In addition, they identified a subpopulation of CD133+CXCR4+ pancreatic cancer cells from human pancreatic cancer tissue samples that localized almost exclusively to the invasive front of the tumor. Postulating that these cells played a role in metastatic potential, CD133+CXCR4+ cells and CD133+CXCR4− cells were orthotopically injected into athymic mice. While both groups of animals evidenced localized tumor development, only mice in the CXCR4+ group demonstrated liver metastases, suggesting an important role for CD133+ CXCR4+ pancreatic CSCs in metastasis formation. This study suggests two forms of cancer stem cells, a localized form and a migratory form. Notably, inhibition of CXCR4 (via both drug and neutralizing antibody) in this tumor model prevented metastatic disease. This finding may have important clinical implications.

At this point, it remains unclear whether there is more than one population of CSCs in pancreatic cancer or the differing results are due to methodological issues. Interestingly, there does appear to be some degree of overlap between CD44+CD24+ESA+ and CD133+ pancreatic cancer cells [90]. While none of above markers appear to selectively characterize a pure population of CSCs, the studies taken together do provide consistent evidence for an enriched CSC population (reviewed in [91]). However, whether CSCs are the *only* cells with tumorigenic potential remains controversial [92, 93].

## 6. EMT Confers a Stem Cell Phenotype and a Metastatic Phenotype

Consistent with the data presented above, CSCs appear to undergo adaptive changes over time as programs are activated that foster invasion and migration. Epithelial-mesenchymal transition (EMT) and the reverse process mesenchymal-epithelial transition (MET) play central roles in organogenesis, and in the context of tumorigenesis, appear to confer CSC properties and drive tumor metastasis (reviewed in [88, 94]). Conventionally, epithelial cells are defined as tightly adherent cells with apical-basal polarity that are organized in adherent sheets via adherens junctions, tight junctions and desmosomes. Typically, epithelial cells are surface barrier cells with secretory functions and remain separated from adjacent tissues by a basal lamina. In contrast, mesenchymal or stromal cells are loosely organized, nonpolarized, migratory cells embedded within extracellular matrix and form the connective tissue or stroma of a given organ. The conversion of epithelial cells to mesenchymal cells involves profound morphological changes including dissolution of cell adhesions, loss of apicobasal polarity and acquisition of migratory and invasive phenotypes. This process appears to be coopted by transformed cells during tumor progression, and interestingly, results in expression of stem-cell markers.

Mani et al. reported that induction of EMT, via ectopic expression of the transcription factors Twist or Snail in immortalized human mammary epithelial cells, leads to acquisition of mesenchymal traits, expression of stem cell markers, and an increased ability to form mammospheres, a property associated with epithelial stem cells [95]. Prior to EMT, these cells express the CD44^low^/CD24^high^ phenotype on the cell surface, corresponding to the phenotype of the majority of cells found in breast carcinomas. Following induction of EMT, the cells undergo conversion to the CD44^high^/CD24^low^ configuration, a profile that is associated with human breast CSCs and normal mammary epithelial stem cells [96]. Injection of the transformed human mammary epithelial cells that have undergone EMT into immunodeficient hosts results in significantly increased tumor formation in comparison to cells lacking either Twist or Snail expression. Morel et al. further extended these findings by noting that activation of the Ras signaling pathway appears to be a crucial event in facilitating the emergence of CD24^low^ cells [97]. Furthermore, microarray analysis of poorly differentiated human tumors associated with an aggressive clinical course were noted to have preferential overexpression of genes normally expressed by ES cells, thus confirming “stemness” as a predictor of poor clinical outcome [98]. Taken together, these findings convincingly demonstrate a link between EMT and the acquisition of stem cell properties.

There is a considerable body of in vitro and in vivo evidence that EMT is centrally involved in the process of metastasis (reviewed in [88, 94]). EMT has been noted to occur at the invasive front of multiple different cancer types including colon carcinoma, breast carcinoma, and papillary thyroid cancers [99–103]. The invasive front of the tumor represents the host-tumor interface and EMT at this border reflects the balance of growth pressures by the tumor body and the tumor periphery. Microarray signatures of EMT have been shown to correlate with aggressive histopathological subtypes of human tumors and inducers of EMT such as SNAIL1, SNAIL2, FOXC, and ZEB1 have been shown to correlate with increased rates of disease relapse and poor overall survival [101, 104–108]. In order to integrate the concepts of CSCs and EMT, Brabletz et al. propose the existence of two cancer stem cell populations, a stationary cancer stem cell and a migratory cancer stem cell, in order to model all aspects of tumor progression [109]. This model has now been preliminarily validated in pancreatic cancer with the identification of two distinct populations of CSCs [90]. 

In order to carefully dissect the contribution of epithelial and mesenchymal states to liver CSC growth and metastasis, Ding et al. established an EMT model of liver cancer. A single CD133+, CD45− liver CSC purified from PTEN^loxp/loxp^/Alb-Cre^+^ mice was expanded and then sequentially transplanted. This population of CSCs have been previously shown to initiate HCC-like and cholangiocarcinoma-like tumors in vivo [110]. With serial rounds of transplantation, two cell populations became apparent; one with epithelial morphology and a second with mesenchymal morphology. Clones of these two cell populations were subisolated and PTEN loss was confirmed, demonstrating that tumor cells were derived from the original PTEN−/− host. Subcutaneous implantation of these cells into nude mice revealed that the mesenchymal subpopulation of cells generated significantly larger tumors than those seen with the epithelial subpopulation or the mixed cell population. Histologically, epithelial cells generated hepatoma like tumors, while the mesenchymal cells formed fibroblastoma-like tumors. The mixed cells formed tumors with both mesenchymal and epithelial cell morphology. Furthermore, in vitro mesenchymal cells were more migratory and invasive than their epithelial counterparts and this was confirmed in vivo by demonstrating their increased metastatic capacity [111].

## 7. Targeting Cancer Stem Cells via Hedgehog Pathway Inhibition

Normal tissue stem cells and CSCs exhibit properties of chemoresistance and radiation-resistance due to their relative quiescence, expression of ATP-binding cassette (ABC) transporters, activated DNA repair systems, and resistance to apoptosis [3]. A growing body of work now supports the idea that chemoradiotherapy spares or even enriches the CSC population within the original tumor, allowing these cells to repopulate the recurrent tumor, despite the fact that the bulk of the tumor has disappeared. In vitro, the CD133+ pancreatic cancer stem cells exhibit significant drug resistance to the chemotherapy agent gemcitabine in comparison to autologous CD133− cells. In vivo, treatment of pancreatic xenografts with gemcitabine also resulted in a significant decrease in tumor volume, but an enrichment of the CD133+ cell fraction, suggesting that CSCs play a role in treatment resistance [90]. Similarly ionizing radiation therapy of human glioma xenografts resulted in an enrichment of the CD133+ glioma CSC subpopulation by fourfold in comparison to untreated controls. Decreased rates of apoptosis, preferential activation of DNA damage checkpoint responses, more efficient repair of DNA damage, and activation of the stem cell renewal pathway, Notch, are some of the mechanisms that underlie the increased survival of CD133+ glioma cells. Pharmacological inhibition of the Chk1 and Chk2 checkpoint kinases as well as the Notch pathway (by gamma-secretase inhibitors) has been shown to overcome the radioresistance of CD133+ glioma cells, suggesting possible therapeutic targets [112, 113]. 

These observations raise the question of whether the CSC represents a new and viable therapeutic target. Some of the most promising therapeutic strategies inhibit SC renewal pathways, such as the Hh pathway, often in combination with conventional cytotoxic agents. The role of hedgehog signaling in pancreatic cancer metastases was investigated by Feldmann et al. using a spontaneously metastasizing xenograft model of pancreatic cancer. These studies demonstrated that while cyclopamine treatment did not significantly inhibit primary tumor growth, rates of distant metastasis to the lung and liver were significantly inhibited by cyclopamine treatment and completely abolished by the combination of gemcitabine and cyclopamine [114]. No histologic distinctions in the tumor tissue were detected between cyclopamine and vehicle-treated animals, but cyclopamine treatment did result in a 3-fold reduction of ALDH-expressing pancreatic cancer cells in vitro. ALDH has been implicated as another putative marker of pancreatic CSCs, and this initial study suggests that inhibition of the hedgehog pathway results in depletion of the pancreatic CSC compartment and is a putative mechanism for metastasis.

Mueller et al. further extended these findings by demonstrating that dual inhibition of the hedgehog pathway and the mTOR pathway in combination with gemcitabine is required to completely eliminates both CD133+ and CD24+CD44+EpCAM+ pancreatic CSC populations in vitro. In contrast, inhibition of the Hh pathway alone in combination with gemcitabine abolished the CD133+CXCR4+ migratory CSC population. Similarly, in a mouse model of orthotopic pancreatic cancer, treatment with cyclopamine, rapamycin (mTOR inhibitor) and gemcitabine was required to fully inhibit growth at the primary site and resulted in a significant overall survival benefit. However, treatment with cyclopamine and gemcitabine resulted in complete inhibition of metastatic activity [115]. Thus, they conclude that dual pathway inhibition is required for complete abrogation of tumorigenic potential. Recently, Olive et al. utilized a mouse model of gemcitabine-refractory, pancreatic ductal adenocarcinoma to demonstrate that treatment with the smoothened inhibitor, IPI-926, alone or in combination with gemcitabine, results in depletion of the desmoplastic stroma and an increase in intratumoral vascular density. In these animals, concentrations of gemcitabine metabolites were augmented by 60% following a ten day pretreatment with IPI-926/gemcitabine. Furthermore, treatment with IPI-926 and gemcitabine (as opposed to either agent alone) resulted in a transient reduction in tumor size at the primary site and significantly inhibited liver metastases, resulting in an overall survival benefit [83]. Their interpretation of these results is that inhibition of the Hh pathway results in increased tumor angiogenesis and stromal collapse, both contributing to enhanced gemcitabine delivery to the pancreatic ductal cells. However, an alternate explanation for these findings is that Hh inhibition depletes the cancer stem cell compartment.

## 8. Conclusions

Cell turnover occurs in all adult organs. Hence, maintenance of normal organ structure and function necessitates appropriate mobilization and differentiation of cells within the stem cell niche of each tissue. Demands on this process increase during adult tissue injury and may unmask defective regulation of pathways, such as Hh and other morphogens that modulate progenitor cell fate. Dysregulation of the Hh pathway has been demonstrated in many types of cancer, including pancreatic and liver cancers. In such tumors, defective Hh signaling has been linked to outgrowth of Hh-responsive cancer stem-initiating cells and stromal elements, suggesting that the Hh pathway might be a therapeutic target.

## List of Abbreviations

Hh: Hedgehog
SC: stem cell
CSC: cancer stem cell
Shh: Sonic hedgehog
Ihh: Indian hedgehog
Dhh: Desert hedgehog
PTCH: Patch
SMO: Smoothened
BCC: basal cell carcinomas
SUFU: Suppressor of Fused
HCC: HCC
Foxa: Forkhead Box A proteins
ES: embryonic stem cells
EMT: epithelial-mesenchymal transition
MET: mesenchymal-epithelial transition
ABC: ATP-binding cassette
